# Atypical Mycobacterium Abscessus Infection in Stable Chronic Lymphocytic Leukemia: A Case Report and Review of the Literature

**DOI:** 10.7759/cureus.20574

**Published:** 2021-12-21

**Authors:** Robin R Rodriguez, Khaled Alhamad, Sohini Ghosh, Eric Bihler, Moses S Raj

**Affiliations:** 1 Medical Oncology, Allegheny Health Network, Pittsburgh, USA; 2 Internal Medicine, Allegheny Health Network, Pittsburgh, USA; 3 Pulmonary and Critical Care, Allegheny Health Network, Pittsburgh, USA; 4 Hematology and Oncology, Allegheny Health Network, Pittsburgh, USA

**Keywords:** oncology, secondary complications, opportunistic infections, mycobacterium abscessus, chronic lymphocytic leukemia

## Abstract

Chronic lymphocytic leukemia is the most common blood cancer in adults. A major cause of morbidity and mortality associated with this cancer stems from opportunistic infections. Similar to many cancers, the inherent effects of battling a raging disease along with the many treatment options causing immunosuppression to lend to the likelihood of obtaining secondary infections.

As it is important for physicians to note the ever-increasing secondary complications, which can manifest in the long-term management of immunosuppressed patients, we present a case of an 86-year-old Caucasian female with stable chronic lymphocytic leukemia who developed intermittent presentation of lung abscesses due to growth of atypical *Mycobacterium *species. With the advent of new treatment options, there has been an increased rate of drug-resistant organisms, lending for the need for more awareness to the severity of these secondary complications and for better options in preventing their occurrence.

## Introduction

Chronic lymphocytic leukemia (CLL) is a very common blood cancer often affecting the older population [1]. Recent advancements have led to better medical management, which has led to increased survival outcomes [1]. As with any cancer, immunodeficiency leads to increased susceptibility to infections. Additionally, with the use of advanced therapies in medical management, there comes a concurrent risk of secondary infection that physicians need to be aware of [2]. Although many of these secondary infections are already well known in the medical community, *Mycobacterium abscessus* is still a challenge in treating [3]. Despite its ability to cause many types of symptoms in the patient population, little has been written in regard to its association with CLL [4]. As it is rapidly becoming known as a highly infectious and multidrug-resistant organism, the increased awareness of possible infection with this organism in the cancer population is paramount.

## Case presentation

This case report is that of an 86-year-old Caucasian female, a former smoker with extensive cardiovascular history, who initially presented eight years ago with a white blood cell count of 14,200 k/mcl with absolute lymphocytosis. She was diagnosed with CLL but was determined to not require therapy at the time of diagnosis. Following initial diagnosis, the patient was lost to follow-up for several years due to moving out of state to Florida where she was followed by another oncologist. While in Florida, the patient’s white blood cell count elevated to 29,000, but she continued to remain asymptomatic and was not started on therapy. Two years later, however, the patient developed anemia with a hemoglobin of 9.8 g/dL and was consequently begun on rituximab at 375mg/m^2^ weekly for four weeks and prednisone twice daily every five days for four weeks. With this therapy, the patient’s anemia became well controlled, with marked improvement of her hemoglobin levels to 13.5 g/dL and her lymphocytosis to a reduced cell count of 4,800 k/mcl.

Following the marked improvement with rituximab, the patient was begun on maintenance therapy, which was subsequently continued upon her return to Pennsylvania. After approximately an additional one year of maintenance therapy of rituximab 90-day cycles, it was determined that this management was no longer necessary as the patient had stable CLL. She has been off maintenance therapy for over one year and has continued to remain asymptomatic with no negative changes in hemoglobin level or blood cell count.

Besides having CLL, the patient has experienced other health issues over the years, including a cerebrovascular accident requiring additional therapy with anticoagulation. She is monitored by neurology along with cardiology for multiple heart issues. Additionally, the patient recently began noting an increased number of infections, most notably acute cystitis. Initially, she was diagnosed with *Enterococcus faecalis* followed two months later by another urinary tract infection (UTI) with predominant *Proteus mirabilis*. She was treated with cephalexin as *P. mirabilis* was found to be resistant to both nitrofurantoin and ampicillin. Due to these recurrent urinary infections, the patient is now also being followed by nephrology and urology.

**Figure 1:**
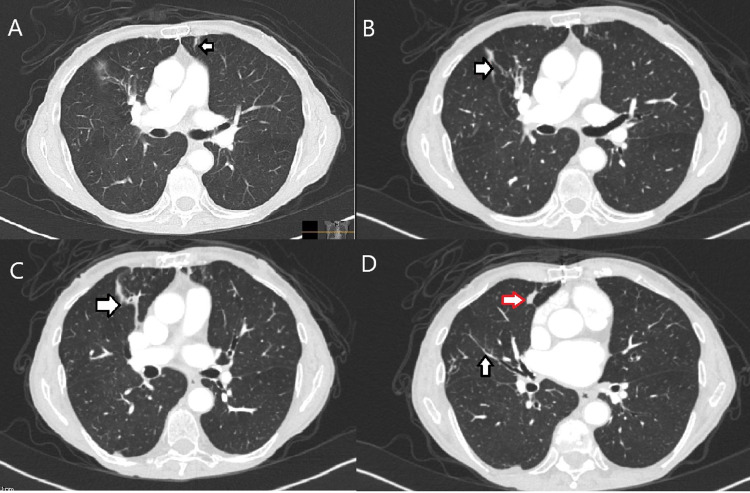
CT restaging scan (A) Left upper lobe nodule. (B and C) Parenchymal opacities with tree/budding opacities demonstrating underlying bronchiectasis. (D). Tree/bud opacities (black arrows) with distal mucus plugging (red arrow).

In regard to CLL, the patient undergoes computed tomography (CT) scans every six months for restaging purposes. On a CT restaging scan, new nodules and masses in the right upper, right lower, and left upper lobes of the lungs were noted alongside evidence of an underlying infectious etiology including pulmonary opacities consistent with bronchiectasis (Figure 1). Pulmonology was consulted following the results of the scan. At that time, due to her smoking history (20 pack-year), malignancy was also considered a differential. The course of action that was determined best was following Fleischner criteria with serial CT scans to be conducted every four months. The first CT scan performed following findings of nodular masses revealed increased consolidation consistent with a worsening infection. Bronchoscopy with bronchoalveolar lavage was therefore performed. Cultures taken during the procedure grew *Mycobacterium abscessus*, although several subsequent bronchoscopies yielded negative stains. The organism was detected via sequencing as DNA probe was unable to yield results. Antimicrobial susceptibility testing (AST) was not reported at that time. Despite the subsequent negative bronchoscopies, secondary cultures taken of expectorated sputum returned positive for *Mycobacterium* negating possible question as to whether initial bronchoscopy finding was a contaminant. Upon multidisciplinary discussion and lack of symptoms, it was determined that the complicated treatment course requiring multiple intravenous (IV) antibiotics for resolution of the bacterium was not warranted. Currently, she is being closely monitored with recent pulmonary function tests continuing to show normal function. She continues to remain asymptomatic at this time and has not been started on any antibiotic therapy for the infection.

## Discussion

CLL is a blood cancer that constitutes 25-35% of all leukemia in the adult population with a median age of 72 years [1,5]. Prognostic factors of CLL include male gender and Caucasian race [6]. Although no specific environmental factors have been definitively related to the development of CLL, studies have illustrated a possible correlation between certain pesticides, rubber, and the formation of CLL [7]. Most patients with CLL are diagnosed incidentally as they are initially asymptomatic [8]. When symptomatic, approximately 5-10% of patients will experience what are known as “B” symptoms [9]. These symptoms are inclusive of the following: weight loss, fever of greater than 100.5 degrees Fahrenheit for more than two weeks, night sweats, and fatigue [9]. Diagnosis of CLL is made with blood counts, smears, and immunophenotyping [8]. The need for treatment is often determined with the use of either Rai or Binet staging along with the presentation of symptoms [8]. These staging techniques help to assess prognostic factors along with management options [8].

Infections constitute a major cause of morbidity and mortality in patients with CLL. CLL patients were found to have inherent defects in both humoral and cell-mediated immunity, in addition to complement deficiency [10]. Also, many agents used in treating patients with CLL have been shown to increase the risk of infection generally and especially opportunistic infections. The introduction of new treatments has resulted in an increase of spectrum of infections in patients with CLL as those treatments have their own impact on the immune system.

In a literature review involving patients who were treated with the purine analog fludarabine, 3.2% of patients developed opportunistic infections [11]. While in a retrospective study involving 368 patients who were treated with ibrutinib, a monoclonal antibody that target Bruton’s tyrosine kinase (BTK), for lymph malignancies, 44% of patients included in the study had CLL. Out of those, 16 patients developed opportunistic fungal infections, with half of those developing invasive aspergillosis [12]. In another retrospective study involving 156 patients who received ibrutinib for CLL as a monotherapy or in conjunction with other agents, 10 patients developed invasive fungal infections [13]. Another class of chemotherapy used in treatment of CLL is BCL-2 inhibitors. In a safety analysis including phase 1 and 2 trials involving 330 patients with relapsed or recurrent CLL who received Venetoclax, opportunistic infections were reported in 11 patients (3.1%), including oral candidiasis, *Aspergillus pneumonia*, *Pneumocystis jirovecii* pneumonia, ocular toxoplasmosis, nocardiosis, herpes pharyngitis, herpes zoster multi-dermatomal, and candida esophagitis [14].

Despite increased risk of opportunistic infections caused by most treatment options in patients with CLL, studies show that patients who were treated with monoclonal antibodies that target CD20, including rituximab, obinutuzumab, and ofatumumab, rarely develop opportunistic infections. In a phase 2 trial including 44 patients treated with first-line single-agent rituximab, no opportunistic infections were reported [15]. However, risk of opportunistic infections increases when rituximab was used in conjunction with other agents. One long-term analysis of CLL patients treated with fludarabine, cyclophosphamide, and rituximab showed that 2% of patients developed an opportunistic infection during the first year of follow-up, with no infections reported beyond the first year of follow-up [16].

Although opportunistic infections are common in patients with CLL, mycobacterial infections appear to be an infection that is extremely uncommon in these patients. Only 13 cases were reported in the literature of CLL or acute lymphocytic leukemia (ALL) patients developing atypical mycobacterial infections [17]. Out of these, only one patient developed the infection prior to treatment for CLL [18]. All other patients received different immunosuppressive agents prior to developing the mycobacterial infection. Additionally, in the cases reported previously, *Mycobacterium abscessus* was only reported in one other case report presenting as lymphadenitis and as a concurrent cutaneous infection four months prior to the patient actually being diagnosed with ALL [4]. Interestingly, in our case, the patient did not develop this opportunistic infection whilst on rituximab or any maintenance therapy, which differs strikingly from many of the aforementioned cases in which these opportunistic infections develop during administration of immunosuppressive agents.

As a non-tuberculous mycobacterium (NTM) member of the species, *abscessus* has proven itself difficult to treat. Presently, it is known for being a widely resistant organism, requiring either multiple drug combinations with the high likelihood of failure in eradicating the bacterium or partial removal of the organ which it infects [19]. Despite more than 170 *Mycobacterium* species, only some of which can cause human infection, *abscessus* has been thought to be one of the most drug-resistant species and unfortunately is one of the most rapidly increasing NTMs in terms of infection rate [20].

Keeping in mind what was known of this bacterium and of our patient, it is easy to understand why it was decided not to treat the infection in its current asymptomatic state. Due to multiple underlying conditions, the asymptomatic presentation of *abscessus* in our case is outweighed by the fragility of our patients in regard to her other conditions. As mentioned, multiple potent drug combinations are not often enough to combat this infection, and resection is often the next option in regard to attempting to eradicate the infection. In situations such as these, the physician needs to make the decision of risk over benefits to best inform the patient of the next suitable course of action.

## Conclusions

With the known likelihood of secondary infections stemming from immunosuppressed states arising from conditions such as cancer, it is important for the physician to be aware of infectious diseases that may pose secondary complications. With the advent of new treatment options for cancer and other related disorders, there has been a wave of infectious organisms that are highly resistant to normal treatment options, making secondary complications even more dangerous than before.

Additionally, these opportunistic infections can take hold in immunocompromised patients even without the addition of suppressive agents due to their already weakened immune state. It is therefore very important for the physician to be able to clinically recognize these opportunistic infections as they arise in chronically ill patients.

## References

[1] Hallek M, Shanafelt TD, Eichhorst B (2018). Chronic lymphocytic leukaemia. Lancet.

[2] Hilal T, Gea-Banacloche JC, Leis JF (2018). Chronic lymphocytic leukemia and infection risk in the era of targeted therapies: linking mechanisms with infections. Blood Rev.

[3] Lee MR, Sheng WH, Hung CC, Yu CJ, Lee LN, Hsueh PR (2015). Mycobacterium abscessus complex infections in humans. Emerg Infect Dis.

[4] Tahara M, Yatera K, Yamasaki K (2016). Disseminated Mycobacterium abscessus complex infection manifesting as multiple areas of lymphadenitis and skin abscess in the preclinical stage of acute lymphocytic leukemia. Intern Med.

[5] Siegel RL, Miller KD, Fuchs HE, Jemal A (2021). Cancer statistics, 2021. CA Cancer J Clin.

[6] Hernández JA, Land KJ, McKenna RW (1995). Leukemias, myeloma, and other lymphoreticular neoplasms. Cancer.

[7] Karakosta M, Delicha EM, Kouraklis G, Manola KN (2016). Association of various risk factors with chronic lymphocytic leukemia and its cytogenetic characteristics. Arch Environ Occup Health.

[8] Hallek M (2019). Chronic lymphocytic leukemia: 2020 update on diagnosis, risk stratification and treatment. Am J Hematol.

[9] Hallek M, Cheson BD, Catovsky D (2018). iwCLL guidelines for diagnosis, indications for treatment, response assessment, and supportive management of CLL. Blood.

[10] Ravandi F, O'Brien S (2006). Immune defects in patients with chronic lymphocytic leukemia. Cancer Immunol Immunother.

[11] Byrd JC, Hargis JB, Kester KE, Hospenthal DR, Knutson SW, Diehl LF (1995). Opportunistic pulmonary infections with fludarabine in previously treated patients with low-grade lymphoid malignancies: a role for Pneumocystis carinii pneumonia prophylaxis. Am J Hematol.

[12] Varughese T, Taur Y, Cohen N, Palomba ML, Seo SK, Hohl TM, Redelman-Sidi G (2018). Serious infections in patients receiving ibrutinib for treatment of lymphoid cancer. Clin Infect Dis.

[13] Nosari A (2012). Infectious complications in chronic lymphocytic leukemia. Mediterr J Hematol Infect Dis.

[14] Reinwald M, Silva JT, Mueller NJ (2018). ESCMID Study Group for Infections in Compromised Hosts (ESGICH) Consensus Document on the safety of targeted and biological therapies: an infectious diseases perspective (Intracellular signaling pathways: tyrosine kinase and mTOR inhibitors). Clin Microbiol Infect.

[15] Sharman JP, Coutre SE, Furman RR (2019). Final results of a randomized, phase III study of rituximab with or without idelalisib followed by open-label idelalisib in patients with relapsed chronic lymphocytic leukemia. J Clin Oncol.

[16] Tam CS, O'Brien S, Wierda W (2008). Long-term results of the fludarabine, cyclophosphamide, and rituximab regimen as initial therapy of chronic lymphocytic leukemia. Blood.

[17] Diaco ND, Strohdach B, Falkowski AL (2019). Psoas abscess due to mycobacterium avium in a patient with chronic lymphocytic leukemia－case report and review. J Clin Med.

[18] Bowen DA, Rabe KG, Schwager SM, Slager SL, Call TG, Viswanatha DS, Zent CS (2015). Infectious lymphadenitis in patients with chronic lymphocytic leukemia/small lymphocytic lymphoma: a rare, but important, complication. Leuk Lymphoma.

[19] Strnad L, Winthrop KL (2018). Treatment of Mycobacterium abscessus Complex. Semin Respir Crit Care Med.

[20] Johansen MD, Herrmann JL, Kremer L (2020). Non-tuberculous mycobacteria and the rise of Mycobacterium abscessus. Nat Rev Microbiol.

